# Sodium-dependent glucose co-transport proteins (SGLTs) are not involved in human glucose taste detection

**DOI:** 10.1371/journal.pone.0313128

**Published:** 2024-11-18

**Authors:** R. Kyle Palmer, Anna B. Nechiporenko, Marc A. Ilies, Marcel Winnig, Stephen A. Gravina, Rashmi Tiwari, Indra Prakash

**Affiliations:** 1 Opertech Bio, Inc., Philadelphia, Pennsylvania, United States of America; 2 Department of Pharmaceutical Sciences and Moulder Center for Drug Discovery Research, School of Pharmacy, Temple University, Philadelphia, Pennsylvania, United States of America; 3 AXXAM SpA, Bresso, Milan, Italy; 4 The Coca-Cola Company, Atlanta, Georgia, United States of America; Zanjan University of Medical Sciences, ISLAMIC REPUBLIC OF IRAN

## Abstract

The sweet taste of saccharides, such as sucrose and glucose, and other sweeteners is known to result from activation of the TAS1R2/R3 receptor expressed in taste receptor cells (TRCs) of the taste bud. Recent reports have suggested the existence of an additional sweet taste signaling pathway for metabolizable saccharides that is dependent on the activity of glucose transporters, especially SGLT1, also expressed in TRCs. We have investigated the potential contribution of SGLT1 to glucose taste signaling in humans. Concentration-response analysis of glucose mediated changes in membrane potential measured in Chinese hamster ovary (CHO) cells transiently expressing the human SGLT1 (hSGLT1) yielded an EC50 value of 452 μM. The SGLT inhibitor phlorizin inhibited the membrane potential response to 10 mM glucose with an IC50 of 3.5 μM. In contrast, EC50 values of 127 and 132 mM were obtained from concentration-response analysis of glucose taste in vehicles of water or 20 mM NaCl, respectively, by rapid throughput taste discrimination with human subjects. Lactisole, an antagonist of TAS1R2/R3, at a concentration of 1 mM completely inhibited taste responses to glucose concentrations of 250 mM and below. Phlorizin (0.2 mM) and the high potency SGLT1-selective inhibitor mizagliflozin (10 μM) failed to inhibit glucose taste detection measured at peri-threshold concentrations in the rapid throughput taste discrimination assay. A Yes/No experiment using the taste discrimination assay revealed that 0.2 mM phlorizin was discriminable from water for some subjects. Taken together the results indicate that agonist activation of TAS1R2/R3 is sufficient to account for all glucose taste without contribution by an alternative SGLT-mediated signaling pathway. Furthermore, the taste of phlorizin could be a confounding variable for studies evaluating a role for SGLTs in taste.

## Introduction

Taste is defined as the sensory process that results from the stimulation of specialized epithelial cells—taste receptor cells (TRCs)—located in the taste buds of the tongue (reviewed in [1]). TRCs are activated when tastant agonist molecules in the oral cavity interact with receptors expressed on their surface. TAS1R2/R3, a heterodimeric class C G protein coupled receptor (GPCR) selectively expressed in subpopulations of TRCs, has been well-established as the principal mediator of gustatory responses to molecules described as “sweet-tasting” by human subjects ([2], reviewed in [3]). Multiple ligand-binding sites have been identified across disparate domains of TAS1R2/R3, accounting for the wide variety of structural classes of molecules among the many TAS1R2/R3 agonists. Saccharide agonists, such as sucrose, sucralose and glucose, bind to a pocket formed by the combined amino termini from each TAS1R protomer, a structure referred to as the “Venus flytrap” (VFT) [4, 5]. A cysteine-rich stretch of residues of the TAS1R3 protomer linking the VFT with the transmembrane spanning domains is critical for agonist activity of protein sweeteners such as brazzein and thaumatin [6, 7], whereas the binding site for cyclamate is within the transmembrane domain of the TAS1R3 protomer [8]. The binding site for lactisole, one of the few molecules identified so far that inhibit responsiveness to sweet tastants by antagonizing TAS1R2/R3, has been shown to overlap with the cyclamate binding site in TAS1R3 [9].

Agonist binding to the TAS1R2/R3 receptor initiates signal transduction through a canonical phospholipase C (PLC) second messenger cascade (reviewed in [3]). Activation of PLCβ2 by βγ subunits of the G protein gustducin generates inositol 1,4,5 trisphosphate (IP3), which in turn binds to and opens a calcium conducting channel, the Type III IP3 receptor (IP3R3) in the membrane of the endoplasmic reticulum. Consequent mobilization of intracellular calcium then results in plasma membrane depolarization by the opening of transient receptor potential melastatin (TRPM) channels 4 and 5. The combined effect of increased intracellular calcium and change in membrane potential finally results in the release of adenosine-5’-triphosphate (ATP) through the Ca2+ homeostasis modulator 1/Ca2+ homeostasis modulator 3 (Calhm1/3) complex [10] into the extracellular space, where it acts as an intercellular mediator to propagate the signal to sensory neurons and other cells within the taste bud (reviewed in [11, 12]).

The association of TAS1R2/R3 with PLC signaling has been confirmed by visualization of component GFP-conjugated proteins [13, 14] and histochemical methods [15, 16], by calcium mobilization assays [17] and by the behavior of mice genetically engineered to be deficient of functional proteins in the PLC pathway. Ablation of the genes that encode IP3R3 [18], PLCβ2 [16], gustducin [19], and each TAS1R2 and TAS1R3 protomer [2] severely impaired but did not entirely eliminate taste-related behaviors to TAS1R2/R3 agonists in mice. The residual behavioral responsiveness toward TAS1R2/R3 agonists was completely abolished in double knockout mice, where both TAS1R2 and TAS1R3 protomers of the heterodimer were absent [2, 20]. Thus it would appear that the intact heterodimer of TAS1R2/R3 is both necessary and sufficient to account for the taste responses to sweet-tasting molecules. Nevertheless, the existence of an alternative sweet taste pathway that functions independently of the TAS1R2/R3 receptor to carry out tastant signal transduction and generate taste responses has been proposed [21–23].

In addition to tastant receptors and their associated signal transduction mechanisms, TRCs also have been shown to express glucose transporter proteins [21, 23] as well as ATP-gated potassium channels [21]. Given the presence of these proteins, taste cells have the potential to act as metabolic sugar sensors, analogous to the glucose uptake mechanisms that are coupled to insulin release in pancreatic β cells (reviewed in [24]). Uptake of glucose, and possibly other metabolizable saccharides, by transporters expressed in TRCs is predominant among those mechanisms proposed as the alternative, TAS1R2/R3-independent sweet taste signaling pathway [25]. According to this hypothesis, once transported into the TRC, glucose is metabolized to yield ATP, which would close the ATP-gated potassium channel and depolarize the cell membrane [21], consequently generating a taste signal.

Sodium-dependent glucose cotransporters, also called sodium-glucose linked transporters (SGLTs), are members of a family of solute carrier proteins encoded by the SLC5 gene (reviewed in [26]). Five of six known SGLTs are symporters, unidirectionally transporting both sodium and glucose across the plasma membrane from the extracellular medium (reviewed in [27]). One SGLT (SGLT3) does not transport glucose, but instead couples to a sodium ion current upon binding to glucose [28]. SGLT1 and SGLT2 are expressed in the renal tubules where they are involved with reabsorption of glucose from the glomerular filtrate. SGLT1 also is expressed in the brush border cells of the intestine where it is responsible for most of dietary glucose absorption (reviewed in [29]). High affinity inhibitors of SGLT1, such as phlorizin and mizagliflozin have been investigated for their potential as therapeutic intervention in hyperglycemia [30, 31]. Little is known of the physiological significance of the remaining SGLTs.

Recently, SGLT1 was identified in TRCs [21, 32, 33], leading to the speculation that SGLTs might have some role in taste signaling. SGLT1 in particular has been proposed to serve as the transporter central to the alternate receptor-independent pathway hypothesis for sweet taste signaling [23]. With co-transport of glucose and sodium, an SGLT1-based mechanism could depolarize a TRC through glucose metabolism and by influx of positive charge carried by sodium.

Uptake by SGLT1 is selective for glucose and galactose, but other saccharides such as fructose and sucrose are not substrates (reviewed in [27]). Any sweet taste signaling mediated by SGLT1 therefore would be limited to glucose and galactose delivered to the tongue as monomers, or possibly liberated from disaccharides, such as sucrose and lactose, or polysaccharides after enzymatic cleavage by orally expressed digestive enzymes [22]. SGLT1 is a high‐affinity, low-capacity transporter for glucose with reported apparent affinities of glucose for hSGLT1 between 0.51 mM and 1.8 mM [34, 35]. Human taste of glucose, on the other hand, is reported to occur at much higher concentrations, with thresholds in the range of 10 mM [36], and suprathreshold concentrations approaching 1 M [37]. These values seem to imply that the taste-active range for glucose is beyond the reach of the concentration range for SGLT1 transport activity.

Nevertheless, some studies investigating mechanisms underlying glucose taste in rodents and humans have presented evidence consistent with sweet taste signaling mediated by an SGLT. For example, adding NaCl to the glucose vehicle has been shown to enhance rates of taste-guided licking of glucose in T1R3 knock out mice [23] and to decrease the glucose taste threshold in humans [36], and in both cases, phlorizin inhibited glucose taste responses. However, the phlorizin concentrations used in both studies (1 mM and 0.2 mM, respectively) are substantially greater than normally would be required to inhibit all SGLTs except the glucose sensor SGLT3 (addressed extensively in Discussion), unless there are appreciable pharmacokinetic barriers to access SGLTs in the taste bud.

We have investigated the potential for an SGLT-mediated mechanism underlying human taste of glucose through experiments with a recombinant hSGLT1 cell-based membrane potential assay and a rapid throughput operant taste discrimination assay for human subjects [38]. Pharmacodynamic parameters of glucose activity in the cell-based assay were compared with the concentration-dependence of human taste responses to glucose in vehicles of water and 20 mM NaCl. We further evaluated the effects of phlorizin in both assays, and also of mizagliflozin and lactisole in the taste discrimination assay. Our results do not support the existence of an alternative, SGLT-mediated mechanism that contributes to the taste of glucose in humans. Instead, glucose taste responses were entirely accounted for by agonist activation of TAS1R2/R3.

## Materials and methods

### Functional expression of SGLT1

Full length human SGLT1 (NP_000334) was generated via gene synthesis (GeneArt^TM^, Thermo Fisher Scientific) and cloned into a pcDNA6 expression vector (Thermo Fisher Scientific).

The cDNAs for hSGLT1 or the empty expression plasmid pcDNA6 were transiently transfected into CHO cells with Opti-MEM I™ using Lipofectamine 2000 (Thermo Fisher Scientific) according to the manufacturer’s protocol. Five to six hours after transfection, the transfection mix was replaced by complete medium (DMEM:F-12 1:1 Mixture w/15mM HEPES, L-Glutamine (BioWittaker) supplemented with 20% FBS (Sigma Aldrich).

Twenty-four hours after transfection, the cells were loaded for 1 hour with a membrane potential dye (Molecular Devices, 1x concentrated, 1.5mg/ml in sodium-free assay buffer consistent of 130 mM Choline Cl, 5 mM KCl, 2 mM CaCl2, 1 mM MgCl, 20 mM HEPES in water at pH 7.4). Changes in membrane potential were recorded by an automated fluorometric imaging plate reader (FLIPR^TETRA^, Molecular Devices) using λexc 510–545 nM / λem 565–625 nM optics. Ligands (Sigma-Aldrich) were dissolved in 200 mM sodium assay-buffer and injected on sodium-free loaded cells, resulting in a final concentration of 100 mM sodium. The kinetic response of the assay was monitored over a period of 5 seconds before compound injection, and for at least 3 minutes after compound injection. All data were collected from at least three independent experiments carried out in quadruplicate.

The obtained fluorescence signals were normalized to the fluorescence of cells prior to the stimulus using DF/F = (F-F0)/F0. Concentration-response curves and EC50 and IC50 values were calculated by nonlinear regression (GraphPad Prism, San Diego, CA). Data are illustrated as % of maximum, where the highest point on the y-axis represents the maximal limit of the glucose concentration-response curve returned from the nonlinear regression, and each other point on the y-axis represents the response as a percentage of this maximum response.

### Liquid chromatography-mass spectrometry (LC-MS) confirmation of phlorizin stability

#### Materials

Phlorizin was purchased from Cayman Chemicals (Ann Arbor, MI). Solvents (HPLC quality) were purchased from Fisher Scientific (Pittsburgh, PA), EMD (Gibbstown, NJ), and VWR International (West Chester, PA), respectively.

#### Methods

The stability of phlorizin under the conditions of storage and handling for human taste testing was confirmed by LC-MS as follows. A stock solution of phlorizin (10 mM) was made by dissolving 6.9 mg phlorizin into 1.58 ml DI water. Two working solutions (200 μM) were made by diluting 20 μl stock solution to a final volume of 1 ml and were incubated either at 4°C or at 25°C. At different timepoints (0, 7, 14 days), samples (10 μl) were taken from the working solutions and were analyzed via liquid chromatography-mass spectrometry (LC-MS) using an Agilent 1200 HPLC-DAD-MS system equipped with a G1315A DAD and a 6130 Quadrupole MS using a ZORBAX SB-C18 column, eluted with H2O (0.1% HCOOH)/MeCN (0.1% HCOOH) 95/5 to 0/100 linear gradient. The injection volume was 1 μl and detection was performed via UV (254 nm) and MS.

### Rapid throughput taste discrimination

#### Materials

Sucrose (Domino Granulated Sugar), was purchased at a local grocery store, D-glucose from Thermo Scientific (Waltham, MA), and NaCl was purchased from Sigma Aldrich (St. Louis, MO). Phlorizin was purchased from Cayman Chemicals (Ann Arbor, MI), and mizagliflozin from Targetmol Chemicals (Wellesley Hills, MA). All tastants and SGLT inhibitors were dissolved in Deer Park Brand Natural Spring Water (Stamford, CT) which also was used for all water trials.

#### Methods

*Apparatus and general methodology for assay*. The rapid throughput operant taste discrimination assay used for all taste experiments described herein were conducted using the TāStation® (constructed and developed according to our design by Biomated Solutions, Randolph, NJ; see [38] for detailed description of the device and methodology). Briefly, the system is comprised of an automated sample delivery device, a programmable electronic pipette with a sterile filter pipette tip (Integra, Hudson, NH), and a laptop with touch-sensitive display (TSD). An interactive software application, in communication with a cloud-based database, coordinates all operations of the device with the execution of the experimental design specified for each subject. The application controls randomized automated presentation of samples, records the subject’s responses, and delivers response-dependent consequences on each trial.

Samples are distributed in a standard 96-well plate which is nested in a 3D-printed tray attached to the surface of an x-y motion table. A 1.5 ml tube filled with water is placed at one end of the tray, serving as a rinse station for the pipette tip in between each trial. The x-y motion table is housed in a cabinet below a vertical support for a z-axis gantry. During the sessions, a cover with a 5 mm diameter hole is placed over the cabinet. The electronic pipette is mounted on the z-axis gantry, held in place by a 3D-printed cradle, with the tip of the pipette directly over the hole in the cabinet lid. On any given trial, the x-y motion table moves the 96-well plate in a randomized pattern to align the center of a single well with the tip of the pipette mounted above. The z-axis gantry lowers the pipette so that its tip enters the well. The pipette draws 200 μl from the well, and then is raised by the z-axis gantry to a position ready for manual removal by the subject. Immediately after removing the pipette, the subject self-administers the 200 μl sample by aiming the tip over the surface of the tongue and pressing the dispense button on the pipette. Subjects are free to apply the sample on an area of the tongue of their choosing.

Subjects are trained through operant conditioning to associate the taste of a stimulus solution with a specific target, centered by specific x,y coordinates in a demarcated area, the response field, on the TSD. The response field is a Cartesian plane, with its origin placed in the upper left corner of Quadrant II. Points along each axis range from the origin to a maximum number of 1.0, thus making the central coordinate pair 0.50, 0.50, and 1.00, 1.00 for the pair of coordinates in the lower right corner of Quadrant IV. The target is designed like a dartboard with three concentric rings around the central coordinates. Upon touching the target designated for the specific taste stimulus, a positive reinforcer in the form of a poker chip immediately appears on the laptop display, accompanied by an audible “cha-ching” onomatopoeia. The poker chip represents actual monetary value, the magnitude of which depends on how close to the central x,y coordinates the subject touches. The greatest chip value ($0.20) results from touches in the “bullseye” (the central circle of the target) and the magnitude of reward decreases progressively as touch responses are made in the second and third outer circles ($0.10 and $0.05, respectively). Touches made outside of the target are penalized by a 10-cent reduction in the subject’s score at that point in the progression through the 96-well trials and are accompanied by a noxious buzzing sound and a 15-second time out. At the conclusion of the final, 96^th^ trial of the session, subjects are immediately paid their cumulative earnings from the taste test by direct deposit into a PayPal account. The weighting of the consequences toward reward almost always results in a positive balance by the session’s end, even under poor performance. In the rare event that the balance is negative, the score is adjusted to zero (no money earned). Subjects are guaranteed $25.00 for every test or training session in which they participated within a month; a check for the total is mailed at the end of the month of their participation.

“Control standard trials” are defined as trials in which the subject must correctly respond on the target designated for the stimulus presented on that trial to obtain the poker chip reward and avoid the penalty. On “test article trials” touch responses made anywhere within the demarcated response field of the TSD are rewarded. All 96 trials of “training sessions” are control standard trials. When subjects reach a criterion of 90% correct responses they advance to “test sessions,” which include both control standard trials and test article trials. The number of training sessions required for achieving the 90% test-ready criterion depends on the difficulty of the taste discrimination central to the experiment and the subject’s demonstrated taste acuity.

#### Methods

*Procedure for current studies*. ***Subjects*.** Informed consents and protocols were reviewed and approved by an independent commercial investigational review board (Advarra, Columbia, MD). Subjects were recruited by word of mouth from the Philadelphia area. Recruitment for the studies began on 14 June 2022 and ended on 1 December 2023. All subjects signed an informed consent, which informed them in writing of the ingredients and SGLT inhibitors in the samples to which they were exposed, and that they would be participating in a taste test that proceeds as a game in which their success depends on their ability to detect and discriminate among different taste stimuli. The informed consent forms were countersigned by the person explaining consent and by an impartial third-party witness. Subjects were excluded who were at the time of testing 1) pregnant or breast feeding, 2) had any major health problems, 3) were known or suspected to have an allergy or other sensitivities to the study materials (or closely related compounds), 4) were taking medications that posed risk of untoward interactions with any of the compounds tested, 5) had any condition or were taking any drug therapy that could affect their sense of smell or the ability to assess the study sample by taste, 6) had any oral symptoms including lesions, sores or inflammation including recent dental work and oral surgery, 7) an adult who lacks capacity to self-consent. A total of 17 subjects (6 male, 11 female) between the ages of 21 and 64 participated in the experiments described herein. All TāStation® studies were conducted in the testing room of Opertech Bio, located in the Pennovation Center of the University of Pennsylvania (Philadelphia, PA). Up to two subjects tested at the same time and were within visual and auditory contact of each other in the testing room, and they were not prohibited from interacting with each other.

Subjects were given verbal instructions on how to handle and operate the pipette and how and where to touch the TSD to record a response. The rest of the procedure was learned through experience with the gamified interactive algorithms. The sequence of events for one complete trial consisted of 1) wash of the pipette tip in the wash tube, 2) randomized movement of the x-y motion table to align a single well beneath the pipette tip, 3) lowering of the pipette by the z-axis gantry and withdrawal of the sample (200 μl) by the pipette from the well, 3) removal of the pipette from the z-axis gantry by the subject and self-administration of the sample to the tongue, 4) replacement of the pipette to the z-axis gantry, 5) recording the subject’s touch-response on the TSD, 6) consequence to the response. Two cups were provided to the subjects, one containing water for a rinse and the other for expectorating the rinse, but rinsing in between trials was not required of the subjects.

Subjects were compensated with cumulative earnings from the gamified training and test sessions by direct deposit into a PayPal account immediately upon the conclusion of a session. Maximal possible earnings from the cumulative score of a session was $19.20, resulting from correctly matching all control standard stimuli to the center of their designated target (plus the full reward value obtained on all test trials of test sessions). In addition to the session earnings, a check summing the total of $25.00 per session for all sessions in which the subject participated in a month was mailed to the subject at the end of that month from Opertech Bio.

#### Concentration response analysis of glucose taste discrimination

These experiments follow the same procedure of [38] for establishing the taste discrimination concentration-response function for sucrose. Here, a range of 8 glucose concentrations was created by serial 2-fold dilutions starting from a maximum concentration of 1 M in aqueous vehicles. The vehicles were water alone, 20 mM NaCl, and 20 mM NaCl+1 mM lactisole. Each of the 8 glucose concentrations was dispensed in volumes of 290 μl into 6 wells of a 96-well plate, and vehicle was dispensed in 24 wells. The remaining 24 wells were reserved for 200 mM sucrose dissolved in vehicle.

Two targets for the taste stimuli were programmed to occur on the x-axis of the response field. A “sweet” target, centered at coordinates 0.75, 0.50 on the x-axis of the response field, was assigned to 200 mM sucrose as a control standard. A “not sweet” target, located at coordinates 0.25, 0.50 along the x-axis, was designated for vehicle as a control standard. The targets were not made visible, but the subjects were informed of their locations in advance of testing. Subjects were instructed to touch the “sweet” target if they detected a sweet taste upon applying the 200 μl sample to the tongue, or the “not sweet” target if no sweet taste was detected. All glucose samples were designated as test articles (touches anywhere in the response field were rewarded), whereas vehicle (water, 20 mM NaCl, or 20 mM NaCl+1 mM lactisole, depending on the experiment) and 200 mM sucrose served as control standards (touch responses had to occur within the radius of the target specifically associated with the control standard). Thus, responses made anywhere in the response field during a glucose trial resulted in a maximum reward, while touch responses on trials of 200 mM sucrose had to occur on the “sweet” target, and on the “not sweet” target for vehicle trials, to obtain a reward and avoid the penalty.

#### Peri-threshold measurement of glucose taste detection by method of constant stimuli (MCS)

These experiments follow the same procedure of [38] for peri-threshold measurement of sucrose taste. Here, glucose was dissolved in aqueous vehicle in concentrations of 20, 40, 60, 80 and 100 mM. The vehicles were water alone, 20 mM NaCl, 0.2 mM phlorizin, 20 mM NaCl+0.2 mM phlorizin, and 20 mM NaCl+10 μM mizagliflozin. Each of the five glucose concentrations was dispensed in volumes of 290 μl into 12 wells of a 96-well plate, and vehicle was dispensed in the remaining 36 wells. As above, subjects were instructed to touch the “sweet” target, centered at coordinates 0.75, 0.50 on the x-axis of the response field, if they detected a sweet taste upon applying the 200 μl sample to the tongue. If no sweet taste was detected, subjects were instructed to touch the “not sweet” target, located at coordinates 0.25, 0.50 along the x-axis. The targets were not made visible, but the subjects were informed of their locations in advance of testing. All trials were control standard trials—responses had to occur on the correctly associated target to receive the award and avoid the penalty.

#### Yes/No detection of phlorizin taste

Water and 0.2 mM phlorizin each was dispensed in volumes of 290 μl into 48 wells of a 96-well plate. Since phlorizin had not previously been categorized as a taste (or other orosensory) stimulus, a target for “any taste other than water” was designated at coordinates 0.75, 0.50 on the x-axis of the response field, and a “water” target at coordinates 0.25, 0.50. Accordingly, if subjects detected any orosensory stimulus present in the background of the water vehicle, they were to touch the “any taste other than water” target, and the “water” target otherwise.

#### Data analysis

The datum for all tests was defined as the number of touch responses made within the boundaries of the “sweet” target (or the “any taste other than water” for the phlorizin Yes/No experiment). The number of target-appropriate responses was divided by the number of trials for a given stimulus solution to obtain response proportions. Day-to-day variability in subject responding was assumed and therefore each subject was tested 6 times for each experiment. The results for each subject were collapsed across all 6 of their tests to give a long-run frequency pattern. The 10 subjects that comprised each experimental cohort were not always the same across all vehicle conditions. Therefore, response proportions were pooled and averaged across subjects in each vehicle condition for the final analyses [39].

For concentration-response analyses, response proportions recorded for each glucose concentration were averaged across all subjects and tests. Curves were fit to the averaged data points by nonlinear regression (GraphPad Prism) and EC50 values, Hill coefficients, and 95% confidence intervals (95%CI) were derived from the curve fit. The curve fitting model used for the regressions was a four-parameter variable slope model following the equation

$$Y=Bottom+\frac{Top-Bottom}{1+10^{\left[(logEC50-X)\;Hill\;Slope\;\right]}}$$

Where *Top* and *Bottom* are plateaus in the units of the *Y* axis, *X* is the log_10_ concentration of agonist in units of molarity.

Statistical determination of differences between pairs of concentration–response functions was achieved by an extra sum-of-squares F test, with the log EC50 selected as the parameter used as the basis for the comparisons (GraphPad Prism). Precision in the calculation of mean values for points used to fit curves in all figures with concentration-response functions is represented by 95%CIs. EC50 values, and attendant 95%CI, for each individual subject are given in Supporting Information (S1 Table).

For MCS peri-threshold measurements, differences between mean proportions of “sweet” responses on trials of glucose and on water trials (Fig 4A) were statistically evaluated by one-way ANOVA with Tukey’s multiple comparisons test (GraphPad Prism). Values for discriminability, *d’*, were calculated for the 20 mM glucose concentration according to the following equation:

$$d^{\prime }=z(H)-z(F)$$

where *z*(*H*) = z-score for the “hit rate, or “true positive rate,” H (the proportion of responses made on the “sweet” target on trials of glucose, or on the “any taste other than water” target on trials of phlorizin) transformed to units of standard deviation (z-score) by the inverse of the normal distribution function, and *z*(*F*) = z-score for the “false alarm rate, or false positive rate,” F (proportion of responses made on the “sweet,” or “any taste other than water,” target on vehicle trials).

Statistical differences among *d’* values were determined by comparison of 95%CIs, which were obtained by multiplying the standard error of the *d’* by 1.96 (the 95th percentile of the standard normal distribution). The *d’* standard error was obtained by taking the square root of the *d’* variance, *var*(*d*′), which was calculated using the equation

$$var\left(d^{\prime }\right)=\frac{H(1-H)}{N_{2}{\left[\phi (H)\right]}^{2}}+\frac{F(1-F)}{N_{1}{\left[\phi (F)\right]}^{2}}$$

where *N*_2_ and *N*_1_ are the number of glucose trials and vehicle trials, respectively, and *ϕ*(*H*) and *ϕ*(*F*) are the values of the standard normal distribution density function for *z*(*H*) and *z*(*F*), respectively [39–41]. Calculated *d’* values, and attendant 95%CI, for each individual subject are given in Supporting Information (S2 Table).

For the Yes/No test for detection of a phlorizin taste, statistically detectable differences between the means of the proportions were obtained by paired t-test (GraphPad Prism), and *d’* values for discriminability of phlorizin were calculated, as described above, for the performance of each individual subject.

Data behind all analyses, means, d’ values, and associated error, and used to construct graphs, and values presented in tables, are given in S1 Data.

## Results

### Recombinant hSGLT1 cell assay

**Fig 1:**
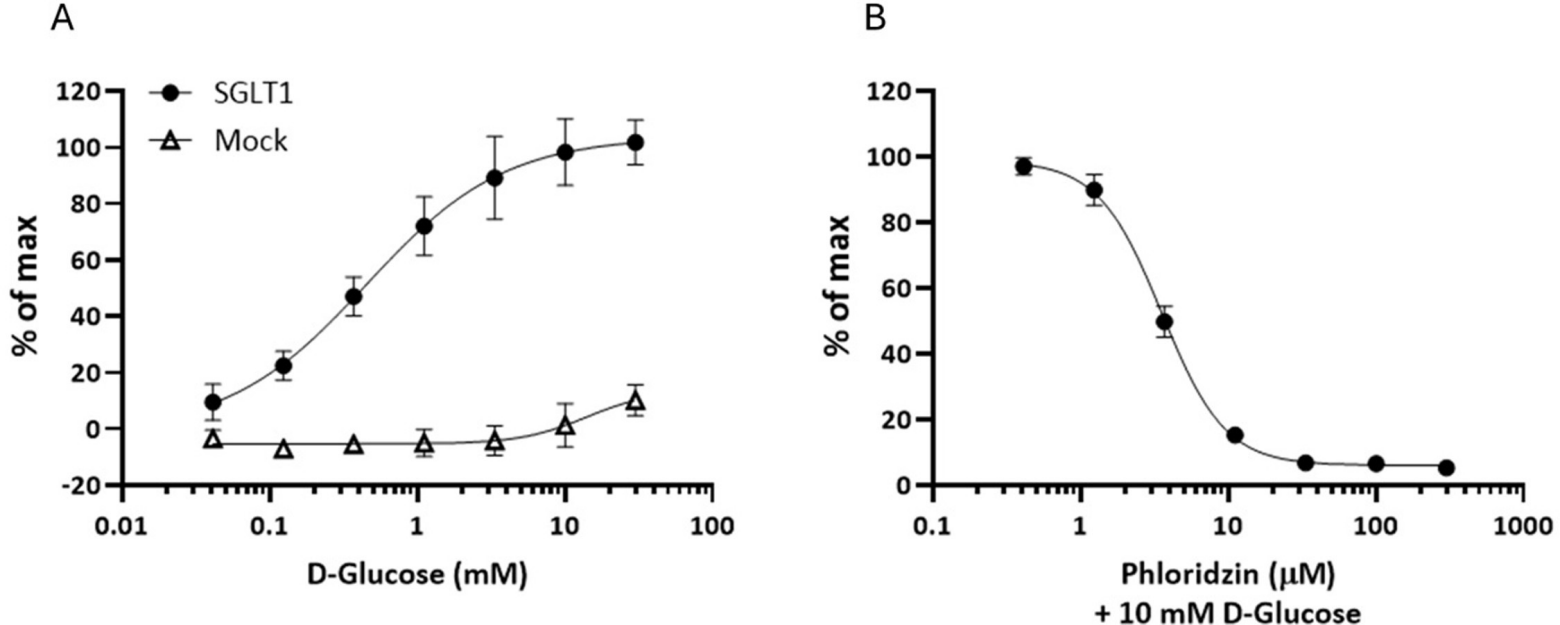
Concentration-dependent activation and inhibition of hSGLT1 measured by membrane potential-dependent changes in fluorescence. CHO cells were transiently transfected with an expression plasmid for hSGLT1 (black filled circles) or an empty expression vector (white empty triangles) and stimulated with increasing concentrations of D-Glucose in the presence of sodium (A) or with decreasing concentrations of phlorizin in the presence of 10 mM D-Glucose (B). Data are illustrated as % of maximum, where the highest point on the y-axis represents the maximal limit of the glucose concentration-response curve returned from the nonlinear regression, and each other point on the y-axis represents the response as a percentage of this maximum response.

Transiently transfected CHO cells expressing SGLT1 were subjected to a concentration-response analysis using D-glucose. The resulting membrane potential read-out revealed a concentration-dependent response with an EC50 value of 452 μM (95%CI = 234–655 μM). Mock transfected CHO cells did not respond to D-glucose (Fig 1A). To further investigate the functionality of hSGLT1, we performed inhibition studies using phlorizin, a known SGLT inhibitor. The inhibitory effect was assessed in the presence of 10 mM D-Glucose, resulting in an inhibitory curve with an IC50 value of 3.5 μM (95%CI = 3.3–3.8 μM, Fig 1B). The TAS1R2/R3 inhibitor lactisole, at concentrations ranging up to 2 mM, had no effect on hSGLT1 activity stimulated by 10 mM glucose (S1 Fig).

### Concentration-response analysis of glucose taste discrimination

To determine the taste discrimination concentration-response function for glucose, and whether 20 mM sodium could impact the function, 10 subjects participated in tests in which 200 mM sucrose and vehicle served as control standards (“correct” responses to these were rewarded and “errors” penalized) and 8 different concentrations of glucose served as test articles (all responses to these were rewarded). Subjects were instructed to make a binary choice between the “sweet” and “not-sweet” targets after pipetting a sample onto their tongue. For both vehicle conditions (all tastants dissolved in water or in 20 mM NaCl), 10 subjects completed 6 tests.

**Fig 2:**
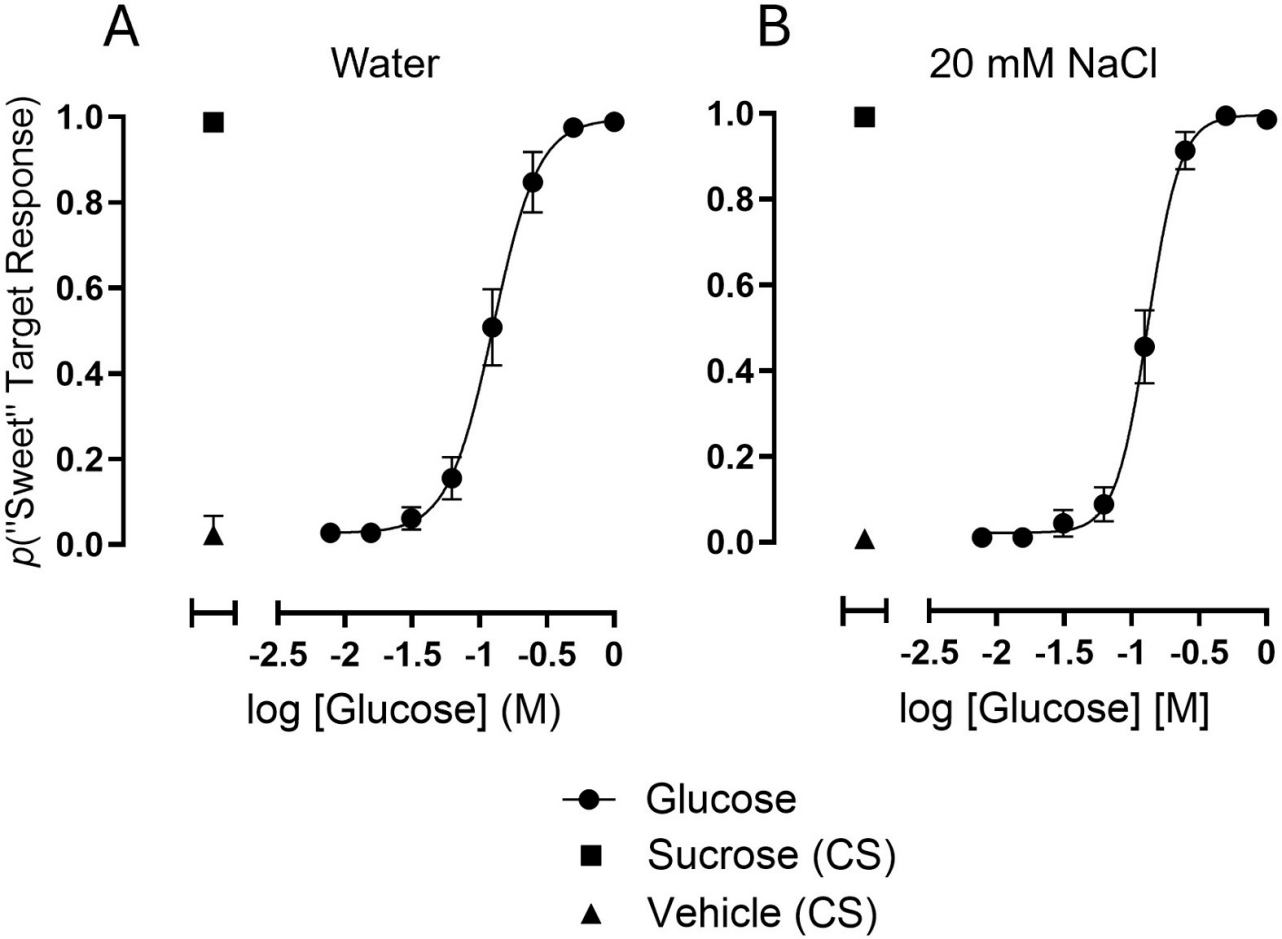
Concentration-response functions for taste discrimination of glucose in a binary “sweet” vs. “not sweet” task. Data are plotted as the proportion of responses that occurred on the target designated for sucrose (the “sweet” target). Error bars are 95%CI. Each concentration of glucose used to generate the functions was presented in 6 replicates per test. All glucose concentrations and the 200 mM sucrose control standard were dissolved either in water (A) or 20 mM NaCl (B) as indicated. Data points were averaged across 60 tests from 10 subjects, each tested 6 times (total of 360 replicates per data point.) Control standards (CS) of 200 mM sucrose and vehicle each were presented on 24 trials per test. Data from control trials are plotted on the left of each graph; the data points for vehicles are the proportions of responses occurring on the “sweet” target (false positives) on vehicle trials. Analysis by nonlinear regression of the data points yielded curve-fit parameters for EC50 of 127 mM (95%CI of 117–138 mM), Hill Slope of 2.58 (95%CI of 2.14–3.15), and an R^2^ coefficient (goodness of fit) of 0.84 when glucose was tested in water (2A); EC50 of 132 mM (95%CI of 126–139 mM), Hill Slope of 3.62 (95%CI of 3.03–4.54), and an R^2^ coefficient of 0.89 when glucose was tested in a vehicle of 20 mM NaCl (2B).

Results from concentration-response analyses performed on data collected across all 60 tests for each vehicle condition are presented graphically in Fig 2.

The overall proportion of “sweet” target responses on control trials of 200 mM sucrose dissolved either in water or 20 mM NaCl was 0.99, and on trials of vehicle were 0.02 and 0.01 for water and 20 mM NaCl, respectively. A curve was fit by non-linear regression to the data points across test article concentrations of glucose to yield potency values of 127 (95%CI = 117–138 mM) and 132 mM (95%CI = 126–139 mM) for the water and 20 mM NaCl vehicle conditions, respectively. The presence of 20 mM NaCl in the vehicle therefore had no detectable impact on the potency of glucose in taste discrimination in the group data. Statistically detectable shifts were observed in the EC50s of 4 individual subjects among the 7 that participated in both test conditions, decreasing for 3 subjects and increasing for 1 subject (see S1 Table). Importantly, these data demonstrate that glucose taste activity occurs in a concentration range that does not overlap with that of human SGLT1 transport activity (compare with the data of Fig 1).

#### Effect of 1 mM lactisole on glucose taste discrimination concentration-response function

**Fig 3:**
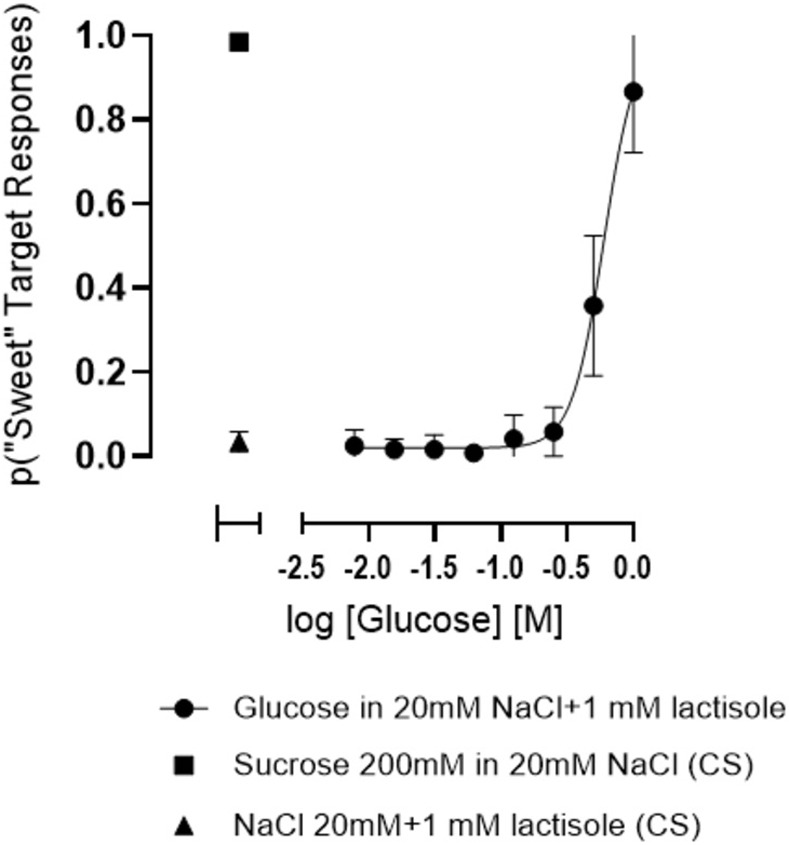
Effect of 1 mM lactisole on concentration-response function for taste discrimination of glucose in a binary “sweet” vs. “not sweet” task. The control standards (CS) were 200 mM sucrose (in 20 mM NaCl) and the vehicle of 20 mM NaCl+1 mM lactisole. All glucose concentrations were dissolved in the vehicle of 20 mM NaCl+1 mM lactisole and were treated as test articles. Data are plotted as the proportion of responses that occurred on the target designated for sucrose (the “sweet” target). Error bars are 95%CI. Each concentration of glucose used to generate the functions was presented in 6 replicates per test. Data points were averaged across 20 tests from 5 subjects, each tested 4 times (total of 120 replicates per data point.) Each CS was presented on 24 trials per test. Data from control trials are plotted on the left; the data point for vehicle is the proportion of responses occurring on the “sweet” target on trials of vehicle (false positives). Nonlinear regression of the data points yielded an approximate EC50 of 598 mM but without 95% confidence intervals.

A concentration-response analysis of glucose taste discrimination in a vehicle of 20 mM NaCl was repeated as before but with the TAS1R2/R3 antagonist lactisole, at a concentration of 1 mM, added to all 8 concentrations of glucose. Since lactisole has been documented to evoke sweet tastes on subsequent trials of water (a “sweet water” response [42]), 20 mM NaCl+1 mM lactisole served as the vehicle control standard; the 200 mM sucrose (in 20 mM NaCl) control standard did not contain lactisole. Five subjects each were tested 4 times. As shown in Figs 3 and 1 mM lactisole greatly impacted the concentration-response function for glucose taste discrimination, essentially eliminating “sweet” target responses at 250 mM glucose and below.

#### Glucose discriminability

**Fig 4:**
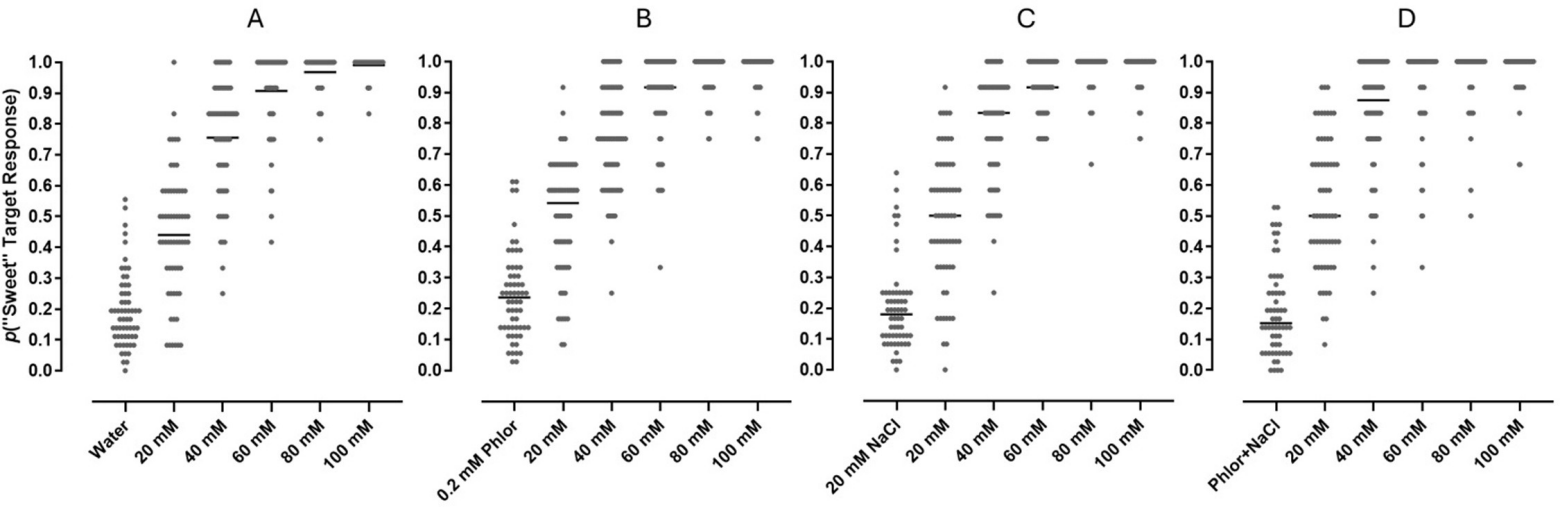
Detection of glucose concentrations below EC50 by an MCS experimental design. Data are plotted as proportions of responses made on the “sweet” target on trials of each stimulus, cumulative from 60 tests (10 subjects tested 6 times) for each condition; means are indicated by horizontal bars. All glucose concentrations were dissolved in vehicles of (A) water, (B) 0.2 mM aqueous phlorizin (Phlor), (C) 20 mM NaCl, (D) 20 mM NaCl+0.20 mM phlorizin. On all trials, “correct” responses were reinforced, and errors were penalized.

An MCS experimental design was used to evaluate the discriminability of 5 concentrations of glucose (20, 40, 60, 80 and 100 mM), dissolved in water, 20 mM NaCl, 0.20 mM phlorizin, and 20 mM NaCl+0.20 mM phlorizin, at peri-threshold detection. Ten subjects each were tested 6 times on the same test design for each of the vehicle conditions. The proportions of “hits” (correctly responding on the “sweet” target on trials of glucose at any concentration, or true positives) and the proportion of false positives (responding on the “sweet” target on trials of vehicle) across all subjects and tests, for all vehicle conditions are shown in Fig 4.

A one-way ANOVA performed on the results from the water vehicle condition (Fig 4A) statistically detected differences in the mean proportions of “sweet” target responses across presentations of the different stimuli (p<0.0001, F (5, 354) = 323.8). Post-hoc analysis revealed differences in responses between water and 20 mM glucose and all other glucose concentrations (p<0.0001), between 20 mM and all other glucose concentrations (p<0.0001), between 40 mM and all other glucose concentrations (p<0.0001), and between 60 mM and 100 mM glucose (p<0.02). These results indicated that the 20 mM glucose concentration would be peri-threshold and also the most sensitive among concentrations as an indicator of the effects of experimental conditions on glucose discriminability.

The overall proportion of hits for 20 mM glucose in water was 0.44 and the false positive rate was 0.20; these values were used to calculate a *d’* of 0.71 (±0.11; Table 1), indicating that 20 mM glucose was discriminable at peri-threshold detection from a background of water vehicle. Addition of 0.2 mM phlorizin to the water vehicle had no impact on discriminability of 20 mM glucose (or on any other concentration of glucose tested; Fig 4B). The *d’* for 20 mM glucose in aqueous 0.2 mM phlorizin was 0.74 (±0.11; Table 1), not statistically different from the *d’* for 20 mM glucose in water.

**Table 1:** Proportions of “sweet target” responses on trials of 20 mM glucose and vehicle and resulting d’ values. Ten subjects were tested 6 times each for all experiments except the 20 mM NaCl+10 μM mizagliflozin (Miz) vehicle condition, in which 8 subjects each were tested 3 times. Each test for all vehicle conditions was composed of 12 trials for each glucose concentration (20, 40, 60, 80 and 100 mM dissolved in vehicle) and 36 trials of vehicle alone. The values given above are the results from the 20 mM glucose trials and vehicle trials. All values given above are cumulative across subjects and tests, and the *d’* and 95%CI were calculated from the cumulative data. Phlz = Phlorizin, *p*(H) = proportion of “hits” (proportion of “sweet target” response on trials of 20 mM glucose), *p*(FA) = proportion of “false alarms” (proportion of “sweet target” responses on trials of vehicle).

Vehicle	*p*(H)	*p*(FA)	*d’* (95%CI)	20 mM Glucose Trials	Vehicle Trials
Water	0.44	0.19	0.71 (±0.11)	720	1260
0.2 mM Phlz	0.48	0.22	0.74 (±0.11)	720	1260
20 mM NaCl	0.48	0.20	0.81 (±0.11)	720	1260
20 mM NaCl+0.2 mM Phlz	0.54	0.19	0.97 (±0.11)	720	1260
20 mM NaCl+10 μM Miz	0.45	0.16	0.85 (±0.11)	288	864

Discriminability of glucose was unaltered by inclusion of 20 mM NaCl (Table 1). The *d’* value for discriminability of 20 mM glucose dissolved in 20 mM NaCl was 0.81 (±0.11). Although slightly greater than the 0.71 *d’* value for the water vehicle condition, the discriminability of 20 mM glucose across the two vehicle conditions was not statistically significant (the 95% CI from the conditions overlapped). For one of the 8 subjects that participated in both water and 20 mM NaCl conditions (subject F1017), the *d’* obtained for 20 mM glucose in 20 mM NaCl was statistically greater than the *d’* prime calculated for that subject’s responses in the water condition (S2 Table).

The addition of 0.2 mM phlorizin to the 20 mM NaCl vehicle did not decrease the discriminability of 20 mM glucose. On the contrary, the *d’* of 0.97 (±0.11, Table 1) for detection of 20 mM glucose in the vehicle of 20 mM NaCl+0.2 mM phlorizin was greater than the *d’* values from all other vehicle conditions, and statistically greater than those obtained in vehicles of either water or aqueous 0.2 mM phlorizin (but not significantly different from glucose in 20 mM NaCl). These data indicate that 20 mM glucose discrimination from background increased when the background vehicle was composed of 20 mM NaCl+0.2 mM phlorizin compared to a background of water.

**Fig 5:**
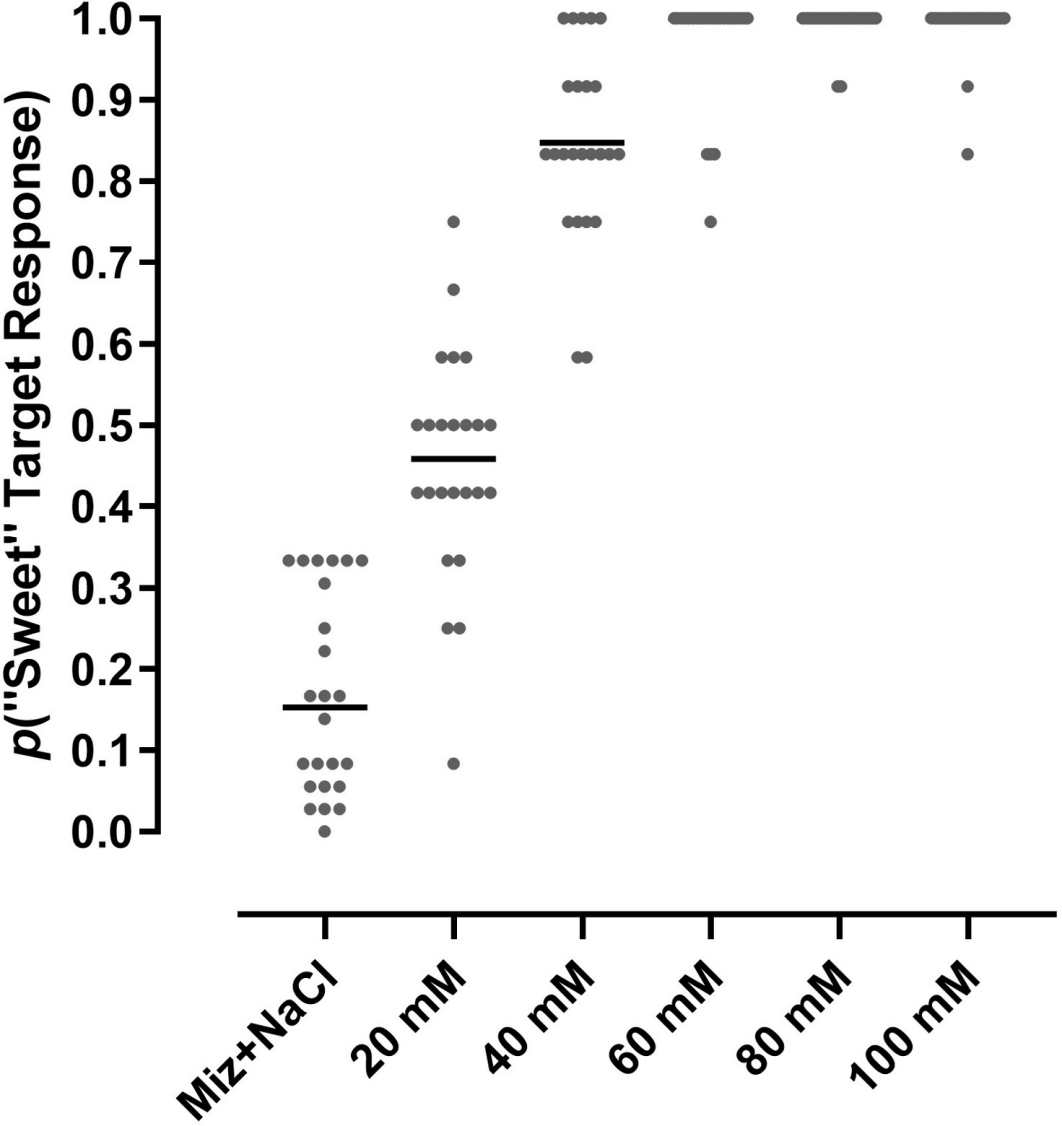
Detection of glucose in the presence of 10 μM mizagliflozin. Data are plotted as proportions of responses made on the “sweet” target on trials of each stimulus, cumulative from 24 tests (8 subjects tested 3 times); means are indicated by horizontal bars. Total number of trials for each concentration of glucose was 288 (12 trials of each concentration per test), and 864 for vehicle (10 μM mizagliflozin; 36 trials of vehicle per test). “Correct” responses were reinforced, and errors were penalized.

Mizagliflozin, an inhibitor with greater potency and selectivity than phlorizin for SGLT1, was evaluated for its potential to affect glucose discriminability. Eight subjects each were tested 3 times using the same MCS design as described above with glucose dissolved in a vehicle of 20 mM NaCl plus 10 μM mizagliflozin. The overall hit rate and false alarm rate were 0.45 and 0.16, respectively, similar to those of all other vehicle conditions (Fig 5).

From these proportions a *d’* of 0.85 (±0.11) was calculated, which was not statistically different from the *d’* value for glucose in the 20 mM NaCl vehicle condition (Table 1). Thus, mizagliflozin, at a concentration that is approximately 400-fold greater than its Ki for human SGLT1 [43], did not decrease discriminability of glucose.

**Fig 6:**
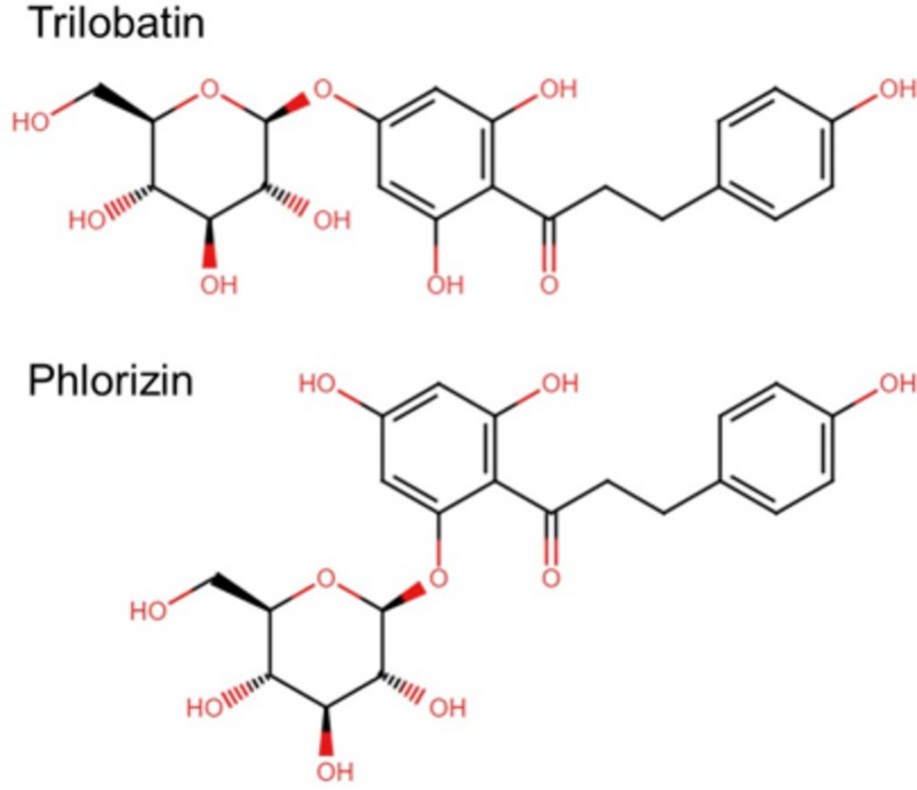
Structural comparison of phlorizin with trilobatin, a compound designated as a FEMA-GRAS-approved taste modifying ingredient.

The failure of the SGLT inhibitors phlorizin or mizagliflozin, both at concentrations greatly exceeding their respective affinity constants for SGLT1, to interfere with peri-threshold detection of glucose indicates that SGLTs are not involved in taste signaling of glucose. The unexpected result of a statistically significant increase in taste detection of glucose when the background vehicle was 20 mM NaCl+0.2 mM phlorizin compared to a background of water suggested that 0.2 mM phlorizin might have intrinsic orosensory properties, potentially adding a discriminatory cue to the vehicle. Indeed, phlorizin is an isomer of trilobatin (Fig 6), a GRAS-approved ingredient (FEMA-4674) with sweetener or taste modifying properties [44].

**Fig 7:**
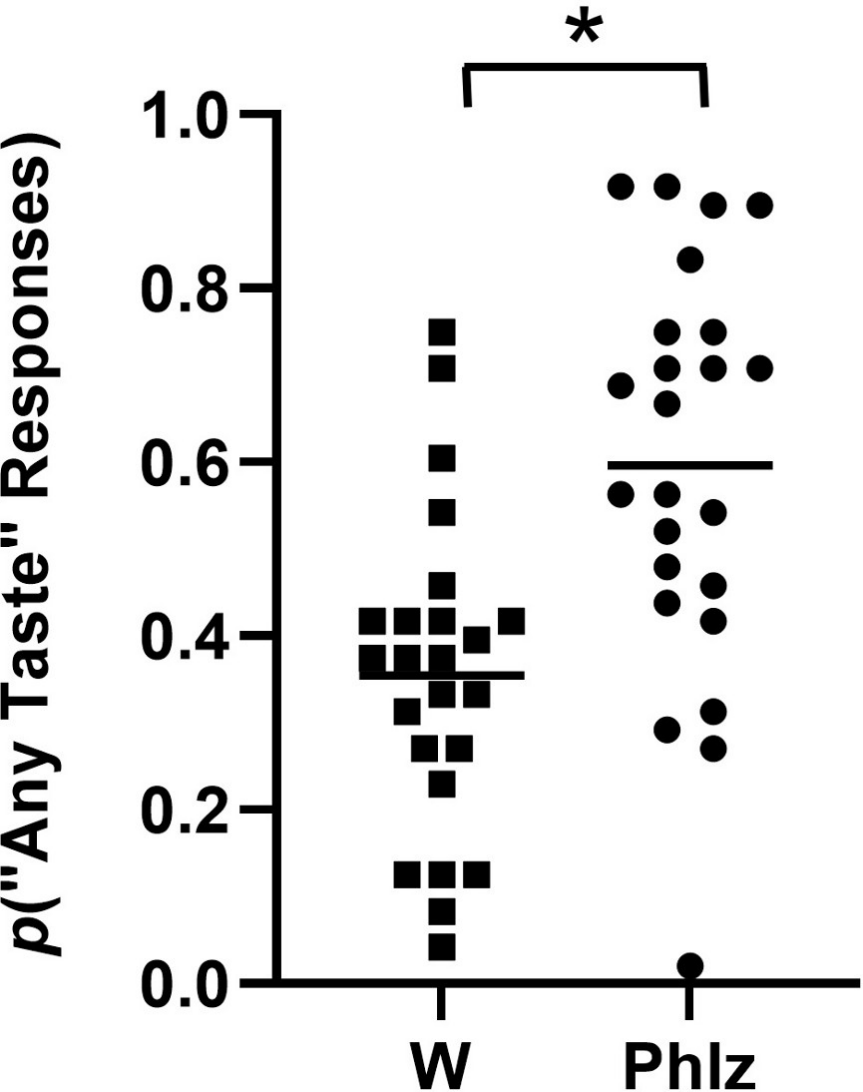
Yes/No test of 0.2 mM phlorizin (Phlz) vs. water (W). Data are plotted as proportions of responses made on the “any taste other than water” target on trials of each stimulus, cumulative from 24 tests (8 subjects tested 3 times); means are indicated by horizontal bars. Total number of trials for 0.2 mM phlorizin and water (48 each per test) was 1,152. “Correct” responses were reinforced, and errors were penalized. Statistically detectable differences between the means of the proportions were obtained by paired t test (* = p<0.0003).

The possibility of 0.2 mM phlorizin being a discriminable stimulus was evaluated by a Yes/No experimental design with 8 subjects, each tested 3 times. Water and 0.2 mM phlorizin were distributed equally in the 96-well plates, so that 48 trials of each were randomly presented to the subjects per test. The results are shown in Fig 7, where the proportion of hits (detection of “any taste other than water” on trials of 0.2 mM phlorizin) and of false alarms (detection of “any taste other than water” on trials of water) recorded from each of the 24 total tests are plotted.

A two-tailed paired t-test performed on the mean hit and false alarm rates (0.60 and 0.35, respectively) yielded a statistical difference (p<0.0003) indicating that, on average, this group of subjects could detect the taste (or other orosensory cue) of 0.2 mM phlorizin from a background of water.

The spread in the data evident in the graph suggested differences in the sensitivities of individual subjects to the taste of phlorizin. Therefore, the *d’* for detection of 0.2 mM phlorizin for each subject was calculated from their hit and false alarm rates over the three tests to determine individual differences; the values are given in Table 2. The results clearly show substantial variation in the ability to detect the taste of 0.2 mM aqueous phlorizin. For example, subjects M1011 (*d’* = 0.00, ±0.31) and M1013 (*d’* = -0.07, ±0.29) were completely insensitive to the taste of 0.20 mM aqueous phlorizin, whereas subjects F1020 (*d’* = 1.43, ±0.34) and F1050 (*d’* = 2.19, ±0.29) readily discriminated phlorizin from the water vehicle.

**Table 2:** Proportions of “any taste other than water” target responses on trials of 0.2 mM phlorizin and water vehicle and resulting d’ values for individual subjects. Eight subjects were tested 3 times each in a Yes/No test between 0.2 mM phlorizin and water. Phlorizin and water were evenly distributed in 96-well plates (48 wells for each), resulting in a total of 144 trials of both stimuli across the 3 tests for each subject. All values given above are cumulative across tests, and the *d’* and 95%CI were calculated from the cumulative data. *p*(H) = proportion of “hits” (proportion of “any taste other than water” target responses on trials of 0.2 mM phlorizin), *p*(FA) = proportion of “false alarms” (“any taste other than water” target responses on trials of water).

Subject	*p*(H)	*p*(FA)	*d’* (95%CI)
F1017	0.57	0.30	0.70 (±0.30)
F1018	0.34	0.28	0.18 (±0.30)
F1010	0.72	0.28	1.16 (±0.31)
F1020	0.88	0.40	1.43 (±0.34)
M1013	0.47	0.50	-0.07 (±0.29)
F1050	0.83	0.11	2.19 (±0.29)
M1011	0.71	0.71	0.00 (±0.31)
M1047	0.33	0.29	0.12 (±0.30)

## Discussion

The primary aim of the current study was to investigate a possible contribution of SGLT activity to the mechanism underlying glucose taste, a proposed alternate sweet taste pathway that operates independently of the TAS1R2/R3 tastant receptor. SGLTs are a family of symporters that move glucose against its concentration gradient into the cell by secondary active transport, coupling glucose transport to the facilitated diffusion of sodium down its gradient into the cell. The first SGLT to be identified was SLGT1, cloned out of cDNA libraries created from rabbit [45] and human [46] intestinal mRNA. More recently, SGLT1 also has been identified in mouse TRCs [21, 32, 33] and consequently has become the main focus of the proposed non-receptor, alternative sweet taste signaling hypothesis [21, 23]. By this proposition, glucose and sodium enter the TRC by SGLT1 co-transport, subsequently stimulating the TRC by some mechanism related to the metabolism of glucose [21], or by influx of positive charge carried by sodium ions sufficient to trigger depolarization.

SGLT1 co-transports sodium and glucose with stoichiometry of 2:1, and when rabbit SGLT1 was expressed in oocytes [47], or when human SGLT1 was expressed in HEK293 cells [35], the transport activity was electrogenic, causing the cells to depolarize upon addition of glucose. It is possible that the activity of an SGLT expressed in taste cells could cause a taste signal by conducting enough Na^+^ ions to depolarize the cell. The electrogenic property of SGLT1 enabled our use of fluorescing membrane potential dye to quantify the pharmacodynamic parameters of SGLT1 ligands in recombinant CHO cells. Concentration-response analysis yielded an EC50 for glucose of 452 μM, with the function saturating as glucose concentration exceeded 3 mM (as would be predicted from receptor occupancy theory). These values are in agreement with those obtained from transport measurements of hSGLT1 reconstituted in liposomes [34], but are about 4-fold more potent than the those from patch clamp measurements in recombinant HEK293 cells expressing hSGLT1 [35]. In either case, our results corroborate the characterization of SGLT1 as a high affinity, low-capacity transporter for glucose.

Phlorizin, a naturally occurring dihydrochalcone and isomer of trilobatin, competitively and potently inhibits the transport of glucose by SGLTs, with greater selectivity for hSGLT2 than for hSGLT1 (apparent affinities of 11 and 140 nM, respectively, [35]). The IC50 values for phlorizin inhibition obtained in our hSGLT1/CHO cell membrane potential assay are consistent with the apparent affinity of 140 nM for hSGLT1 measured by patch clamp in hSGLT1-expressing HEK293 cells [35]. These measurements then set up the next question of whether the range of concentration-dependence of SGLT1 activity corresponds to the range for taste.

We have applied rapid throughput taste discrimination technology and methodology to rigorously characterize the entire taste-active concentration range for glucose, as well as evaluate the ability of human subjects to detect glucose taste at peri-threshold concentrations. This assay was used previously to do the same for sucrose and other TAS1R2/R3 agonists and was thoroughly validated according to standards set by the National Institutes of Health for assay development, and importantly, the results from those studies were consistent with receptor occupancy theory. It is furthermore important to note that desensitization of taste responses did not occur across the 96 trials of a test session [38].

The capability of our rapid throughput taste discrimination assay to randomly present multiple samples in replicate within single test sessions enabled robust, quantitative characterization of the entire concentration-response function for glucose taste. Following self-administration of a sample to the tongue, the subjects were constrained to one of two possible response options—touching either the “sweet” or “not sweet” target—within a demarcated field on a touch-sensitive display. This binary response output created a frequency pattern interpretable as the probability of a “sweet” target response occurring on a trial of any given concentration of glucose or vehicle. The resulting data points supported curve fits by non-linear regression. The probability of a “sweet” response was lowest for trials of glucose at concentrations of 31.25 mM (i.e., the third lowest of the range) and below, corresponding to peri-threshold for detection (compare with Fig 4A and the *d’* for 20 mM glucose, Table 1). The function rapidly accelerated as the glucose concentration exceeded 31.25 mM and saturated as the concentration approached 500 mM. Curve fitting yielded an EC50 of 127 mM when the vehicle was water. This result demonstrates that the range of taste activity for glucose is beyond the capacity of glucose transport by SGLT1 (Fig 1), which would be at a maximum before reaching 10 mM. Since sodium ions are co-transported with glucose by SGLT1, the experiment was repeated with 20 mM NaCl as the vehicle to determine whether the presence of sodium would impact the concentration-response function. The EC50 obtained for glucose taste discrimination with a vehicle of 20 mM NaCl, 132 mM, was similar to the EC50 from the water vehicle condition, and thus no effect of sodium across the taste-active concentration range for glucose was statistically detected on the pooled data set. Closer inspection of the response patterns of individual subjects indicated that for a few, 20 mM NaCl might have had a small, but statistically detectable impact on the glucose taste discrimination concentration-response function, either increasing or decreasing glucose potency (S1 Table).

Lactisole has been demonstrated to inhibit the activation of the human TAS1R2/R3 receptor by saccharide and other agonist sweeteners [9, 48, 49]. Accordingly, lactisole has been demonstrated to inhibit the sweet taste of glucose and other saccharides in human subjects. For example, 250 ppm lactisole (approximately 1 mM) completely eliminated the perceived sweetness intensity of glucose at concentrations of 8.37% (approximately 440 mM) and below [37]. More recently [50], the detectability of 75 mM glucose measured by a triangle discrimination task was reduced from a *d’* value of 1.77 to a *d’* of 0 (completely indiscriminable from the blanks) when 1.4 mM lactisole was added to the samples. In agreement with these studies, 1 mM lactisole effectively blocked the taste of glucose in our concentration-response analysis of glucose taste discrimination—essentially all responses on the “sweet” target were eliminated at concentrations 250 mM and below (i.e., responses on those trials of glucose occurred on the “not sweet” target, Fig 3). A recent study [36], however, reported that 2 mM lactisole raised the threshold of glucose taste to 41.8 mM, which implies that glucose above this concentration would be readily detectable, and therefore stands in contrast to previously published work as well as our current results.

Given the pharmacodynamic limits of SGLT activity and the concentration-response function for glucose taste, any contribution to taste signaling by SGLTs could only occur (if at all) at the very lowest levels of glucose taste detection. The rapid throughput taste discrimination assay therefore was applied to determining the effects of sodium and SGLT inhibitors on peri-threshold detection of glucose. Using an MCS experimental design, vehicle (36 trials per test) and five concentrations of glucose, ranging from 20 to 100 mM (12 trials each per test), were randomly presented to subjects to quantify peri-threshold detection of glucose taste. The response output again was binary, with subjects recording “sweet” target and “not sweet” target responses on independent sets of coordinates in the touch-sensitive display. In the peri-threshold measurements the contingencies of reinforcement were different from those of the concentration-response test. In the concentration-response analysis, there were two control standards (on these trials correct responses were rewarded, errors penalized)—a maximally effective stimulus (200 mM sucrose) and vehicle—responses to all other stimuli were rewarded no matter which target was selected. In the peri-threshold test, all stimuli were treated as control standards—correct responses rewarded, errors penalized—and this had the effect of shifting the context to the lower range of concentrations (below EC50), thus magnifying the response scale.

Under a presumption that taste detection at peri-threshold concentrations is inherently variable, each subject was tested six times across multiple days to accommodate day-to-day fluctuations in the measurements that might occur. Taste threshold measurement was thus treated as a long-run response frequency pattern appearing in cumulative trials of vehicle (216 trials) and each glucose concentration (72 trials) across the 6 tests of each subject. Under these conditions, 20 mM glucose was observed to be near the limit of discriminability from the background of a water vehicle (Fig 4A).

Signal Detection Theory (SDT) is a conceptual and quantitative framework that provides a powerful statistical methodology for evaluating performance in sensory discrimination tests [41]. By applying SDT statistics to datasets comprised of proportions of binary responses, the discriminability between two stimuli can be quantified and represented by a single value, the *d’*, ranging from 0 (no discrimination) to limits around 4 (perfect discrimination). The *d’* value of 0.71 (±0.11; Table 1) indicates a peri-threshold discriminability of 20 mM glucose from water, a determination consistent with the glucose detection threshold of 13.3 mM generated by the staircase 2-alternative forced choice (2-AFC) method of Breslin et al, 2021 [36].

Peri-threshold indices of detection should be acutely sensitive to manipulations that influence the mechanisms underlying glucose taste. If SGLTs are contributing to taste signaling generated by low glucose concentrations, then inhibitors of SGLT activity should measurably affect peri-threshold glucose detection. With an apparent affinity for hSGLT1 of 140 nM [35], phlorizin at a concentration of 0.2 mM should be more than sufficient for a competitive inhibition of glucose taste if mediated by hSGLT1 at 20 mM glucose, a view supported by our cell-based assay results in which phlorizin inhibited responses to 10 mM glucose with an IC50 of 3.5 μM. However, when tested in a background vehicle of water, 0.2 mM phlorizin had no impact on the discriminability of glucose (Table 1). Perhaps it could be argued, however, that the concentration of sodium in saliva, or more specifically, in the immediate extracellular space of SGLTs in the taste bud, was not sufficiently high to drive SGLT-mediated glucose transport and reveal its involvement in taste. Indeed, 0.2 mM phlorizin was reported to increase the threshold of glucose detection in a 2-AFC test when the tastant vehicle was 20 mM NaCl [36]. Therefore, peri-threshold measurement of glucose discriminability, in the presence and absence of 0.2 mM phlorizin, was repeated with a vehicle of aqueous 20 mM NaCl. The *d’* for 20 mM glucose in 20 mM NaCl appeared to be slightly greater than that obtained in the water vehicle (0.81±0.11 and 0.71±0.11, respectively). But since the confidence intervals calculated for these values overlap, the apparent increase is not supported statistically. Inspection of *d’* values calculated for individual subjects similarly suggests a trend toward greater discriminability of glucose in a vehicle of 20 mM NaCl, and for one subject, the increase was statistically detectable (S2 Table).

More importantly however, when 0.2 mM phlorizin was added to the NaCl vehicle, discriminability of 20 mM glucose did not decrease, but instead, increased to a *d’* of 0.97 (±0.11). Though suggestive of a counterintuitive enhancement of glucose discriminability, the difference in *d’* between the 20 mM NaCl and 20 mM NaCl+0.2 mM phlorizin vehicle conditions did not achieve statistical significance. In no case among the *d’* values calculated for individual subjects that participated across all vehicle conditions was there a significant decrease in discriminability of glucose in the presence of 0.2 mM phlorizin (S2 Table).

Mizagliflozin is another SGLT inhibitor, with greater affinity and selectivity than phlorizin for hSGLT1. Inhibitor constants of 27 nM for mizagliflozin inhibition of human SGLT1 and 8170 nM for human SGLT2 have been reported [43]. As with phlorizin, mizagliflozin at a concentration of 10 μM (approximately 400 times its apparent affinity for hSGLT1) had no impact on 20 mM glucose discriminately when tested in the 20 mM NaCl vehicle (Table 1; compare Fig 5 with Fig 4C).

Both phlorizin and mizagliflozin are competitive inhibitors of glucose transport by SGLTs. The Cheng-Prusoff equation [51] is the standard for predicting the relationship between the affinity of inhibitor (Ki) and the IC50 (the concentration of inhibitor required to produce 50 percent inhibition of the competing substrate, in this case glucose as an agonist). The equation is as follows:

$$IC50=K_{i}\left[1+\left(\frac{\left[A\right]}{KD_{A}}\right)\right]$$

Where [*A*] is the agonist concentration and *KD*_*A*_ is the affinity of the agonist.

Using the affinity values for phlorizin and glucose at hSGLT1 of 0.14 μM and 1.8 mM, respectively (values provided by [35]), and a glucose test concentration of 20 mM, the above equation yields an IC50 for phlorizin of 1.7 μM. If using the glucose affinity value of 0.51 mM [34], then the IC50 of phlorizin would be 5.6 μM against 20 mM glucose. If using the EC50 value of 452 μM obtained in our membrane potential assay as an estimate of glucose affinity for hSGLT1, then the IC50 for phlorizin against 20 mM glucose would be 6.3 μM. Thus, the 200 μM concentration of phlorizin used in our human taste test, as well as those of others [36], was more than sufficient to eliminate activity of SGLT1, the only SGLT known to be expressed in mammalian taste cells. Applying the Cheng-Prusoff equation to the SGLT1-selective inhibitor mizagliflozin (Ki = 27 nM, [43]), the IC50 against 20 mM glucose would be 1.2 μM if the affinity of glucose for SGLT1 is 452 μM, and 0.3 μM if glucose affinity is 1.8 mM.

Repeating the calculation for phlorizin inhibition of the other SGLTs will be useful if evidence of their expression in mammalian taste cells appears in future publications. The expected IC50s can be calculated by using affinity values for phlorizin and glucose, respectively, from the literature for hSGLT2 (0.011 μM and 4.9 mM [35]), hSGLT3 (120 μM and 19 mM [52]), hSGLT5 (1.7 μM and 10 mM for α-methyl-d-glucose instead of D-glucose [53]), and hSGLT6, also known as SMIT2 (76 μM and 30 mM [54]). The resulting IC50s for phlorizin vs. 20 mM glucose for the SGLT2, SGLT3, SGLT5 and SGLT6 are 0.06 μM, 246 μM, 5 μM, and 127 μM, respectively. Phlorizin Ki for hSGLT4 was not reported by Tazawa et al [55] but inhibitory potency appeared to be 10-fold lower than for hSGLT1 in that study, and therefore the IC50 vs. 20 mM glucose would be approximately 20–60 μM. Given these calculations, it is expected that 200 uM phlorizin should have noticeable, if not dramatic impact on taste detection of 20 mM glucose if any of the known SGLTs contribute to glucose taste signaling, with the exception of SGLT3 (but SGLT3, along with SGLT2, is absent in mouse circumvallate [33]).

There are some important distinctions to note regarding the technology of our rapid throughput taste discrimination assay from the methodology of traditional taste testing, one of the most obvious being the difference in sample volumes. The volume of the stimulus samples used in our study was 200 μl, whereas sample volumes often range in the 10s of milliliters for traditional taste tests. The use of such relatively small volumes could raise questions over whether they are adequate for activating all mechanisms underlying a taste signal. For example, it might be speculated that a taste signal arising from metabolism of glucose following SGLT transport could take longer to develop compared to a receptor mediated pathway and might be missed unless larger volumes of sample were held in the mouth. But since the non-metabolizable SGLT substrate α-methyl-D-glucopyranoside (MDG) is effective at eliciting a taste response [36], metabolism of glucose is not necessary for the putative alternative sweet taste signaling pathway. If an SGLT was involved, co-transport of sodium would have to be the event that depolarizes taste cells, an action that would be expected to occur as rapidly (if not more so) than agonist activation of a GPCR. Furthermore, the *d’* values we obtained for 20 mM glucose detectability, indicate near- but above-threshold detection (hence our use of the term “peri-threshold), an observation consistent with the absolute threshold determinations of others [36] using 10 ml volumes of glucose solutions.

Another argument could be raised that a sample volume of 200 μl might be insufficient to reach an SGLT that could be spatially separated from the location of TAS1R2/R3. However, if human tongues are similar to mouse tongues, SGLTS are known to be expressed in taste cells that also express TAS1R2/R3, the “sweet” committed taste cells. Since the 200 μl sample immediately evokes a taste response to glucose, the stimulus solution can’t have far to go to reach an SGLT. A larger volume would therefore have no advantage at reaching the target, even if the SGLT was expressed on the basal end of the taste cell. We must conclude that our use of 200 μl sample volumes is sufficient to capture the activity of any known signaling pathway involved in sweet taste.

When conducting in vivo taste experiments, it is important to establish whether the pharmacological tools used to probe putative underlying signaling mechanisms exhibit any intrinsic taste properties. For example, an inhibitor of one mechanistic target that also has tastant agonist properties at another could introduce additional sensory cues, a complication for interpretating any observed effects on discrimination behavior. In earlier research efforts exploring the mechanism of salt taste, there was some suspicion that the sodium channel blocker amiloride might carry an intrinsic aversive taste that would impact rodent behavioral responses to solutions of NaCl. Conditioned taste aversion experiments proved that rats detected little if any taste of amiloride and thereby it could be safely concluded that amiloride’s behavioral effects were due to direct interference with epithelial sodium channels in the taste signaling mechanism [56]. NaCl is the quintessential representative stimulus for the basic taste of “salty.” NaCl at a concentration of 20 mM is detectable in human taste discrimination [38] and therefore will provide an additional sensory cue to subjects in a glucose taste discrimination. Thus, the task of detecting glucose in a vehicle of 20 mM NaCl should not be considered equivalent to the task of detecting glucose in water—the two background conditions are different, potentially impacting the outcome of discriminability measurement.

Phlorizin is a structural isomer of the dihydrochalcone trilobatin (Fig 6), a FEMA-designated flavor ingredient with a detectable taste at 100 ppm (0.29 mM; [44]). The taste activity of trilobatin suggests that phlorizin might also be taste-active at similar concentrations. In our automated, rapid throughput version of the Yes/No experiment, we found that on average, 0.2 mM phlorizin was discriminable from a background of water (Fig 7). The range of *d’* values given in Table 2 indicates that some subjects detected the presence of phlorizin whereas others were completely insensitive. Phlorizin, with its intrinsic taste, could add to the orosensory properties of water, and presumably with 20 mM NaCl, to create a more complex background from which to detect low concentrations of glucose. The additional taste of phlorizin (at least for some subjects) could impact discriminatory behavior in tests of glucose threshold or peri-threshold taste detection, particularly if there are no consequences to the “choice.” Reinforcing correct choices and penalizing incorrect choices immediately upon responding, as was done in the currently presented experiments, could improve discrimination of the taste cue of interest from the other cues present in a complex background. On the other hand, if there are no consequences to the choice in a discrimination test, as is the case in most traditional measurements of taste thresholds, any similarities in stimulus properties between the tastant of interest and components the vehicle background could negatively impact discriminability.

Verbal instructions given to subjects on how to perform a discrimination task potentially can influence the outcome of the measurement [57], particularly if there are no consequences to the response. Placing contingencies of reinforcement on the choice behavior minimizes the impact of any bias that might ensue from the presence of verbal cues in the instructions. Although we instructed subjects to select the “sweet” or “non-sweet” targets for some experiments, contingencies of reinforcement were in place that rewarded correct responses and penalized incorrect choices regardless of whether the subject was responding on the basis of a verbally dictated “sweet” cue. Under the contingencies of reinforcement in an operant Yes/No taste detection design, subjects will be shaped to respond to any detectable orosensory cue.

The alternative sweet taste pathway hypothesis rests heavily on the observations of residual behavioral and neural responding to glucose and other sweeteners when only one protomer of the TAS1R2/R3 receptor is lost, as in T1R3 knock out mice [2, 23, 58, 59]. But the weight of this evidence is diminished by the fact that knocking out both protomers of the TAS1R2/R3 receptor completely eliminates taste sensitivity to sweeteners, including glucose [2, 20]. This fact alone implies that the activity of the intact, functional heterodimer TAS1R2/R3 receptor accounts for all of the sweet taste stimulated by saccharides and non-nutritive sweeteners. Furthermore, not all studies agree that residual sensitivity to glucose remains when the gene for T1R3 is ablated. For example, T1R3 knockout mice were unable to discriminate 2.0 M glucose from water after multiple discrimination training sessions [60]. Most recently, T1R3 knockout mice again were shown to be insensitive to 2.0 M glucose in taste discrimination, even with the addition of 10 mM NaCl to the glucose solution [61]. Finally, the ability to entirely block sweet taste of glucose (as was demonstrated here and elsewhere) with the TAS1R2/R3 receptor antagonist lactisole, supports the notion that all taste signaling of sweet molecules, whether metabolizable or not, is explained by agonist action on TAS1R2/R3. Even if an alternative pathway was needed to complete our understanding of sweet taste signaling, a mechanism based on an SGLT would not be a good candidate, since transport activity already is saturated by glucose concentrations that are just nearing detection thresholds for taste. The presence of glucose transporters and ATP-dependent potassium channels in taste cells instead suggests other glucose-mediated physiological processes that are not directly related to, or are independent of, the process of generating a detectable taste stimulus.

## Conclusion

Here, human subjects were presented with many trials of glucose at multiple concentrations, in the presence or absence of the co-transported SGLT substrate sodium, as well as the SGLT inhibitors phlorizin and mizagliflozin, and the TAS1R2/R3 antagonist lactisole. In contrast to recently reported results [36], we found no evidence of involvement of an SGLT in detection of glucose taste by human subjects under the conditions of testing with a rapid throughput taste discrimination assay. We further have established the concentration ranges for hSGLT1 transport activity and for glucose taste, and the two do not overlap. The TAS1R2/R3 inhibitor lactisole completely eliminated taste responses to glucose below 250 mM. Thus, all of the taste detection of glucose by human subjects in our study is accounted for by activity of the TAS1R2/R3 receptor, with no evidence of contribution by an SGLT-mediated mechanism. Finally, any study investigating the mechanisms underlying taste detection must establish whether the pharmacological agents used also impart a taste stimulus that could interfere with clear interpretation of results, especially when testing at threshold detection levels.

## Supporting information

S1 Fig Lactisole does not inhibit hSGLT1 activity measured by membrane potential-dependent changes in fluorescence. CHO cells were transiently transfected with an expression plasmid for hSGLT1 and stimulated with 10 mM glucose in the presence of increasing concentrations of lactisole. Changes in membrane potential-dependent fluorescence were recorded by FLIPR. Data are illustrated as % of maximum change in membrane potential-dependent fluorescence stimulated by 10 mM glucose in the absence of lactisole.
(TIF)

S1 Table Individual subject EC50s and 95%CI from concentration-response analysis of glucose taste in vehicles of water and 20 mM NaCl measured by rapid throughput taste discrimination. Concentration-response functions were evaluated for statistically detectable differences for those subjects who participated in both conditions by an extra sum-of-squares F test, with the log EC50 selected as the parameter used as the basis for the comparisons (GraphPad Prism). NS = not significant, ??? indicates failure to compute the limit.
(DOCX)

S2 Table Individual subject d’ values for discriminability of 20 mM glucose in vehicles of water, 0.2 mM phlorizin, 20 mM NaCl, and 20 mM NaCl+0.2 mM phlorizin. Taste responses to glucose (20, 40, 60, 80 and 100 mM) and water were recorded in a rapid throughput taste discrimination assay using a method of constant stimuli experimental design. Phlz = phlorizin, p(H) = proportion of hits (responses made on the “sweet” target on trials of 20 mM glucose), p(FA) = proportion of false alarms (responses made on the “sweet” target on trials of vehicle), 95%CI = 95% confidence interval.
(DOCX)

S1 Data (XLSX)
