# Supplementary figures and images for: Sodium-dependent glucose co-transport proteins (SGLTs) are not involved in human glucose taste detection
Source: PLoS One. 2024 Nov 18;19(11):e0313128. doi: 10.1371/journal.pone.0313128 (PMC11573166; doi:10.1371/journal.pone.0313128)

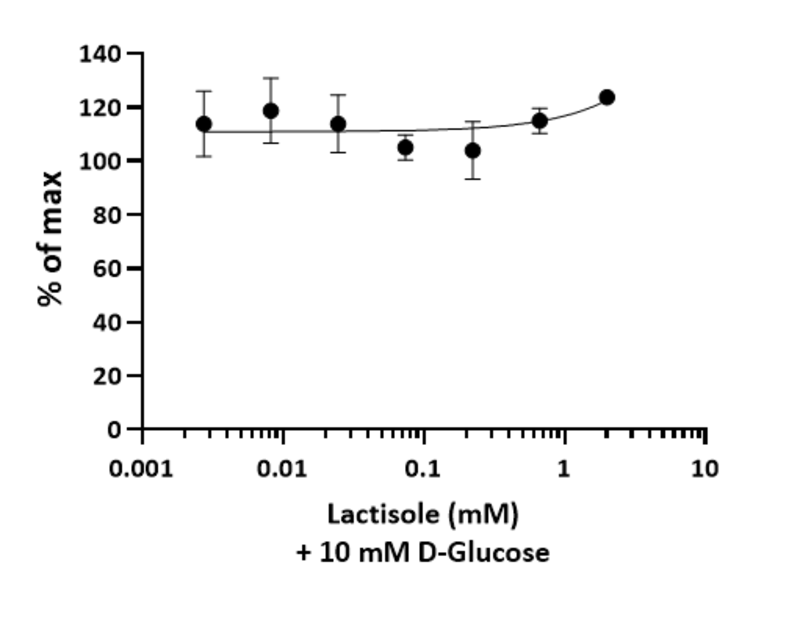

Supplement: S1 Fig — CHO cells were transiently transfected with an expression plasmid for hSGLT1 and stimulated with 10 mM glucose in the presence of increasing concentrations of lactisole. Changes in membrane potential-dependent fluorescence were recorded by FLIPR. Data are illustrated as % of maximum change in membrane potential-dependent fluorescence stimulated by 10 mM glucose in the absence of lactisole. (TIF) [file pone.0313128.s001.tif]
