# Supplementary material for: Sodium-dependent glucose co-transport proteins (SGLTs) are not involved in human glucose taste detection
Source: PLoS One. 2024 Nov 18;19(11):e0313128. doi: 10.1371/journal.pone.0313128 (PMC11573166; doi:10.1371/journal.pone.0313128)
Supplement: S1 Table — Concentration-response functions were evaluated for statistically detectable differences for those subjects who participated in both conditions by an extra sum-of-squares F test, with the log EC50 selected as the parameter used as the basis for the comparisons (GraphPad Prism). NS = not significant, ??? indicates failure to compute the limit. (DOCX) [file pone.0313128.s002.docx]

|  | Water Vehicle | | 20 mM NaCl Vehicle | |  |
| --- | --- | --- | --- | --- | --- |
| Subject ID | EC50 | 95%CI | EC50 | 95%CI | Significance |
| F1017 | 135 | 111 - 163 | 138 | 119 - 160 | NS |
| M1013 | 119 | 100 - 145 | 85 | 75 - 97 | p=0.006 |
| M1011 | 154 | 139 - 173 | 135 | ??? - 145 | NS |
| F1018 | 71 | 64 - 80 | 98 | 87 - 110 | p=0.001 |
| F1020 | 126 | 113 - 141 | 116 | 100 - 132 | NS |
| F1058 | 296 | ??? - 363 | 124 | 117 - 133 | p<0.0001 |
| F1050 | 130 | 111 - 151 | 97 | 83 - 113 | p=0.03 |
| F1015 | 141 | 112 - 186 |  |  |  |
| F1023 | 79 | ??? - 89 |  |  |  |
| F1057 | 140 | 100 - 205 |  |  |  |
| F1056 |  |  | 188 | 162 - 218 |  |
| M1047 |  |  | 180 | 150 - 216 |  |
| F1067 |  |  | 250 | ??? |  |

|  |  |
| --- | --- |
|  |  |
|  |  |
