# Supplementary material for: Sodium-dependent glucose co-transport proteins (SGLTs) are not involved in human glucose taste detection
Source: PLoS One. 2024 Nov 18;19(11):e0313128. doi: 10.1371/journal.pone.0313128 (PMC11573166; doi:10.1371/journal.pone.0313128)
Supplement: S2 Table — Taste responses to glucose (20, 40, 60, 80 and 100 mM) and water were recorded in a rapid throughput taste discrimination assay using a method of constant stimuli experimental design. Phlz = phlorizin, p(H) = proportion of hits (responses made on the “sweet” target on trials of 20 mM glucose), p(FA) = proportion of false alarms (responses made on the “sweet” target on trials of vehicle), 95%CI = 95% confidence interval. (DOCX) [file pone.0313128.s003.docx]

|  | Water | | | 0.2 mM Phlz | | | 20 mM NaCl | | | 20 mM NaCl + 0.2 mM Phlz | | |
| --- | --- | --- | --- | --- | --- | --- | --- | --- | --- | --- | --- | --- |
| SUBJECT ID | **p(H)** | **p(FA)** | d’ (95%CI) | **p(H)** | **p(FA)** | d’ (95%CI) | **p(H)** | **p(FA)** | d’ (95%CI) | **p(H)** | **p(FA)** | d’ (95%CI) |
| F1015 | 0.56 | 0.42 | 0.34 (±0.34) | 0.57 | 0.42 | 0.37 (±0.34) | 0.69 | 0.46 | 0.61 (±0.35) | 0.54 | 0.34 | 0.51 (±0.34) |
| F1017 | 0.31 | 0.18 | 0.40 (±0.36) | 0.43 | 0.15 | 0.87 (±0.36) | 0.58 | 0.17 | 1.18 (±0.35) | 0.53 | 0.10 | 1.34 (±0.37) |
| F1018 | 0.33 | 0.17 | 0.54 (±0.36) | 0.47 | 0.13 | 1.06 (±0.36) | 0.42 | 0.28 | 0.38 (±0.34) | 0.54 | 0.06 | 1.62 (±0.39) |
| F1020 | 0.50 | 0.18 | 0.91 (±0.35) | 0.51 | 0.17 | 1.00 (±0.35) | 0.40 | 0.08 | 1.17 (±0.38) | 0.71 | 0.13 | 1.70 (±0.37) |
| M1013 | 0.19 | 0.12 | 0.33 (±0.40) | 0.39 | 0.19 | 0.58 (±0.35) | 0.57 | 0.19 | 1.07 (±0.35) | 0.42 | 0.20 | 0.63 (±0.35) |
| M1011 | 0.42 | 0.23 | 0.52 (±0.35) | 0.57 | 0.25 | 0.86 (±0.34) | 0.46 | 0.15 | 0.92 (±0.35) | 0.81 | 0.40 | 1.11 (±0.37) |
| F1010 | 0.50 | 0.13 | 1.15 (±0.36) | 0.42 | 0.18 | 0.72 (±0.35) |  |  |  |  |  |  |
| F1012 | 0.50 | 0.23 | 0.73 (±0.34) | 0.54 | 0.21 | 0.90 (±0.35) |  |  |  | 0.47 | 0.14 | 1.02 (±0.36) |
| F1023 | 0.63 | 0.06 | 1.83 (±0.39) |  |  |  |  |  |  |  |  |  |
| M1015 | 0.47 | 0.23 | 0.68 (±0.34) | 0.61 | 0.22 | 1.05 (±0.35) |  |  |  |  |  |  |
| F1050 |  |  |  | 0.31 | 0.25 | 0.17 (±0.35) | 0.40 | 0.15 | 0.80 (±0.36) | 0.65 | 0.23 | 1.14 (±0.35) |
| F1057 |  |  |  |  |  |  | 0.39 | 0.21 | 0.53 (±0.35) |  |  |  |
| F1058 |  |  |  |  |  |  | 0.65 | 0.11 | 1.64 (±0.37) |  |  |  |
| M1062 |  |  |  |  |  |  | 0.28 | 0.20 | 0.24 (±0.36) |  |  |  |
| M1046 |  |  |  |  |  |  |  |  |  | 0.36 | 0.12 | 0.82 (±0.37) |
| M1047 |  |  |  |  |  |  |  |  |  | 0.40 | 0.21 | 0.57 (±0.35) |
